# The influence of deprivation and pregnancy planning on multiple health behaviour change before pregnancy: a secondary analysis of the Scottish Maternal and Infant Nutrition Survey

**DOI:** 10.1186/s12884-026-09709-7

**Published:** 2026-08-05

**Authors:** Hannah Welshman, Stephan U Dombrowski, Aileen Grant, Vivien Swanson, Sinéad Currie

**Affiliations:** 1 Faculty of Natural Sciences, University of Stirling, Stirling, UK; 2 Faculty of Kinesiology, University of New Brunswick, Fredericton, Canada; 3 Aberdeen Health Determinants Collaboration, Aberdeen City Council, Marshall College, Aberdeen, UK

**Keywords:** Preconception, Survey, Health behaviour change, Pregnancy planning, Deprivation

## Abstract

**Background:**

Engaging in multiple, positive health behaviours prior to pregnancy improves health of adults and their future offspring for generations. Socio-demographic factors are often associated with health behaviours and outcomes, e.g. people from more deprived areas are more likely to engage in health risk behaviours during pregnancy and have highest rates of unplanned pregnancies, ultimately perpetuating health inequalities. This secondary analysis aimed to explore how relative deprivation and pregnancy planning status influence multiple health behaviour change before conception.

**Methods:**

Secondary analysis was conducted on The Scottish Maternal and Infant Nutrition Survey (SMINS), a large-scale survey distributed to pregnant women across Scotland in 2017. Demographic data were collected including a measure of deprivation using the Scottish Index of Multiple Deprivation (SIMD), along with pregnancy planning status (London Measure of Unplanned Pregnancy, LMUP) which includes one question self-reporting behaviour changes prior to pregnancy (e.g. folic acid, smoking, alcohol and diet). A composite score for multiple behaviour change was generated ranging from zero to four where a higher score indicated greater health behaviour changes.

**Results:**

Of the 2,523 responses, 78% of pregnancies were classed as planned (LMUP 10–12), 19% as ambivalent (LMUP 4–9) and 3% unplanned (LMUP 0–3). There was a significant effect of deprivation on mean composite score for health behaviour change (*p*<.05) where behaviour change scores in the most deprived quintile, SIMD 1 (M=0.93, SD = 1.08), were significantly lower than every other SIMD quintile. There was a significant effect of pregnancy planning on multiple behaviour change (*p*<.001) where multiple health behaviour change was significantly higher in the planned category (M = 1.5, SD = 1.06) than the ambivalent category (M = 0.23, SD = 0.60) and the unplanned category (M = 0.04, SD = 0.26). Overall change in multiple health behaviours was low. Across all participants, there was a higher degree of psychological planning compared to behavioural planning.

**Conclusions:**

There is sub-optimal engagement in multiple health behaviour change before conception. However, this is particularly low among non-planners and people living in areas of higher deprivation. Individuals psychologically prepare for pregnancy more than they behaviourally prepare. Lack of behavioural preparation can have negative impacts on the physical health of women and babies.

## Background

An individual’s health and behaviours before conception can have long lasting effects on pregnancy outcomes and the health of future offspring [1]. For example, maternal overweight or obesity before and during the early stages of pregnancy are associated with adverse birth outcomes including macrosomia (birth weight of greater than 4500 g) [2] which increases risks of childhood obesity.

Engaging in multiple health behaviours prior to pregnancy such as folic acid supplementation, healthy eating, smoking cessation and reducing/stopping alcohol consumption not only improves health of adults but has also been recognised as aiding reduction of future childhood morbidity and mortality [3]. Importantly, research has shown that high risk women who consume 5 mg of folic acid per day before pregnancy reduce their baby’s risk of neural tube defects by approximately 85% [4]. However, nine in 10 women enter pregnancy with at least one potentially modifiable risk factor for pregnancy and birth complications such as smoking, obesity and lack of folic acid supplementation [5]. Hence, it is important to understand engagement in these modifiable health behaviours prior to pregnancy.

Research from England, Belgium and USA has suggested that socio-demographic factors and pregnancy planning status are associated with engagement in preconception health promoting behaviours [5–7]. Socio-demographic factors such as socio-economic deprivation have been found to influence smoking behaviour before pregnancy where people who lived in areas of greater deprivation were more likely to be smokers prior to pregnancy [8]. These factors perpetuate health inequalities (health differences between social groups) which continue to grow in Scotland [9]. For example, there is a gap of 24 years healthy life expectancy between those living in the least and most deprived areas of Scotland [10]. Furthermore, maternal smoking rates in the most deprived areas of Scotland are 11 times higher than in the least deprived areas in Scotland [9]. These factors need to be further explored and tackled to reduce health inequalities and improve health outcomes for future generations.

Socio-demographic factors are associated with pregnancy planning where more unplanned pregnancies are reported in those who live in the most deprived areas of the UK [11]. Furthermore, there is complexity in the relationship between pregnancy planning and multiple health behaviour change. For example, women in Denmark who reported planning their pregnancies were more likely to engage in some health promoting behaviours before conception such as supplementing with folic acid and quitting smoking. However, 20% of these planners still engaged in binge drinking in early pregnancy, compared to 30% of non-planners [12]. Similar results were found in the USA where women who intended to conceive were not any more likely to engage in preconception health promoting behaviours compared with non-planners, except for folic acid supplementation [13]. This highlights the complexity of the term ‘pregnancy planning’ which often ranges from unplanned to ambivalent to planned [14] and indicates potential difference between pregnancy planning and preparation, as found by Maas et al. [15] as well as variance in types of pregnancy preparation.

Understanding how people plan and/or prepare for pregnancy may be helpful to understand why there is variation in engagement in health behaviours and health behaviour change (i.e. behaviourally preparing for pregnancy) between planners and non-planners and within planners themselves.

Multiple health behaviours influence preconception and long-term health and multiple behaviours are all recommended throughout the preconception period [16]. However, it is important to consider these multiple behaviours together, rather than on their own, many health behaviours (and risk factors) are inter-linked [17]. Additionally, to tackle health inequalities, it is important to consider how deprivation and pregnancy planning status influence multiple health behaviour change. These findings could help to shape future preconception health interventions and campaigns.

This secondary analysis aimed to explore how deprivation and pregnancy planning status influence multiple behaviour change before conception using a national data set from Scotland (Scottish Maternal and Infant Nutrition Survey, SMINS, 2017) [18]. The research questions were:

1. What is the association between deprivation and multiple health behaviour change prior to conception?
2. Is pregnancy planning associated with multiple health behaviour change prior to conception?
3. How do behavioural and psychological pregnancy planning relate to multiple behaviour change?

## Methods

### Design and setting

This study reports a secondary analysis of data from the SMINS which was a large-scale survey authorised by the Children and Families Directorate of the Scottish Government. The survey was designed to investigate health and nutrition for women prior to, during and after pregnancy as well as for infants. The antenatal part of the survey was distributed to women who were over 20 weeks pregnant across Scotland between 1st May 2017 and 30th June 2017. The survey measured pregnancy planning using the six item London Measure of Unplanned Pregnancy (LMUP, 14), as well as demographic factors including relative deprivation (measured using the Scottish Index of Multiple Deprivation, SIMD).

### Participants

Survey packs were provided to eligible patients by staff from maternity services at routine antenatal appointments when women were over 20 weeks pregnant. For this secondary analysis, respondents who answered the antenatal section of the survey were included.

### Survey

Surveys were completed on paper or online. Postcodes were collected and mapped onto SIMD to categorise relative deprivation. SIMD ranks areas within Scotland into quintiles using to domains which contribute towards deprivation including income, education, health, employment, crime, housing and access to services. SIMD quintiles range from the most deprived (SIMD 1) to the least deprived (SIMD 5) areas of Scotland.

The LMUP [14], was used as a reliable and valid measure to assess the extent to which a person has planned their pregnancy. Questions include “Just before I became pregnant. □ I intended to get pregnant, □ my intentions kept changing, □ I did not intend to get pregnant” and “Just before I became pregnant… □ I wanted to have a baby, □ I had mixed feelings about having a baby, □ I did not want to have a baby”. The LMUP has six questions, each individual scores as zero, one or two, with question scores summed to produce a total LMUP score, a 0–12 scale. A score of 10–12 is designated to the “planned” category, a score between 4 and 9 is termed the “ambivalent” category and a score 0–3 is the “unplanned” category. Hence, the LMUP evaluates stance (internal feelings towards pregnancy), context (external environment and personal circumstances) and behaviour (actions taken before pregnancy) to generate a picture of how planned a pregnancy was [14]. 

The authors of this study disaggregated the LMUP and identified three items which measure a behavioural aspect to planning (e.g. asking whether an individual has taken specific action to prepare) and three items which measure a psychological and emotional aspect of planning (e.g. feelings about the timing of conception). Hence, this secondary analysis used the LMUP in accordance with the validated LMUP guidance (RQ1-2) as well as separating the six questions into two categories to explore health behaviour change in relation to behavioural and psychological components (RQ3).

Data on preconception health behaviour change was collected through item six of the LMUP where participants were asked “Before you became pregnant, did you do anything to improve your health in preparation for pregnancy? (tick all that apply) took folic acid, stopped or cut down smoking, stopped or cut down drinking alcohol, ate more healthily, sought medical/health advice, took some other action (please describe) or I did not do any of the above before my pregnancy. A composite score for multiple behaviour change was generated from this question. Of the seven response options, four were included for use in this secondary analysis (took folic acid, stopped/ cut down smoking, stopped/ cut down drinking, ate more healthily). Excluded items included “Sought medical/health advice” which was not included due to ambiguity regarding what the advice sought was and whether it was related to pregnancy. “Took some other action” was also excluded as it was unclear what the action was and if it was related to pregnancy and preconception health. The last item which was excluded was “I did not do any of the above for my pregnancy” as it did not add additional information regarding specific health behaviours.

To generate the composite score for multiple behaviour change, a score of one was given when items were answered with yes and a score of zero was given when items were answered with no. Hence the range spanned from a total score of four where yes was selected for each item, indicating greater change in health promoting behaviours to zero for no change.

### Data analysis

**Fig. 1:**
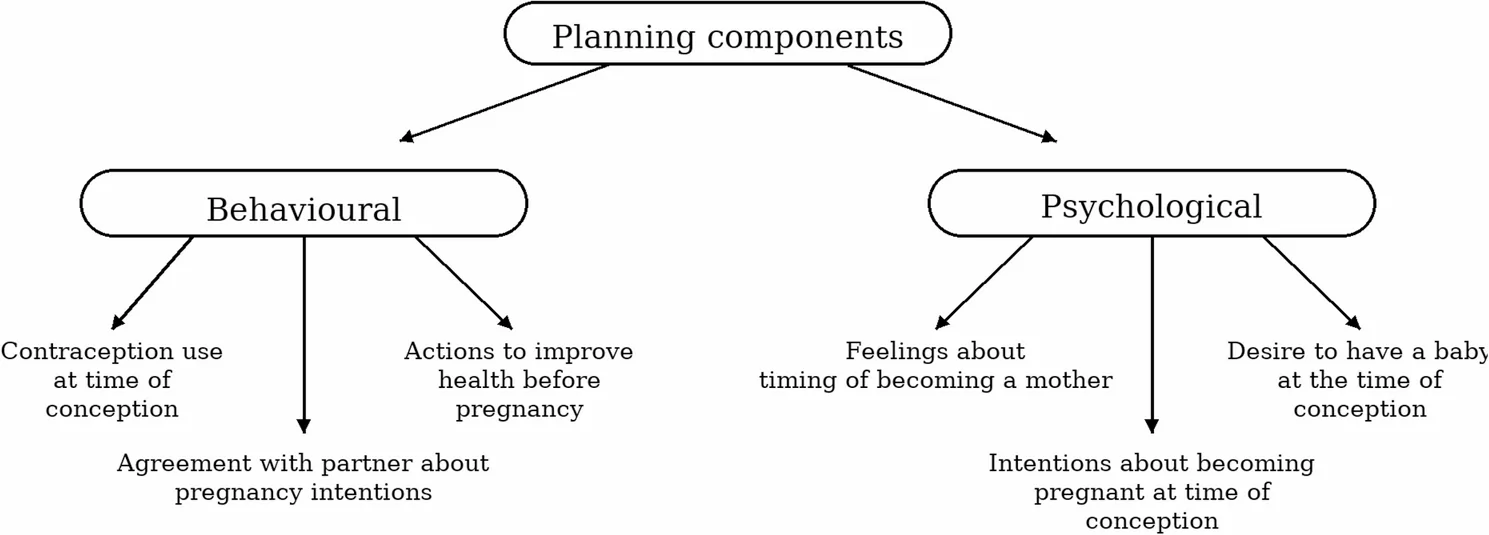
Generation of behavioural and psychological planning components using LMUP questions

To explore pregnancy planning in more detail, the authors generated two variables to consider items from the LMUP from a behavioural and a psychological perspective (Fig. 1). This approach is novel, exploratory and not part of the LMUP scoring guidance hence the behavioural and psychological categories are not psychometrically validated. The behavioural component was constituted of three LMUP items which involve action such as discussions with partners and taking specific action to optimise health or choose to cease contraceptive use. The psychological component included items which concern thoughts, feelings and intentions regarding pregnancy rather than actions. Once the two components were created for this analysis, the maximum score for each component (behavioural planning and psychological planning) was six (range zero-six).

For research question 1, a one-way ANOVA was conducted to explore the impact of SIMD quintiles (range 1–5) on multiple behaviour change with a Bonferroni adjustment conducted as a post-hoc test. Research question 2 also required a one-way ANOVA to be conducted to explore the impact of LMUP categories (planned, ambivalent, unplanned) on multiple behaviour change, with the Bonferroni adjustment conducted as a post-hoc test. To determine if multiple behaviour change differed between behavioural planning and psychological planning (research question 3), two one-way ANOVAs were conducted with a Bonferroni adjustment used as a post-hoc tests.

## Results

**Fig. 2:**
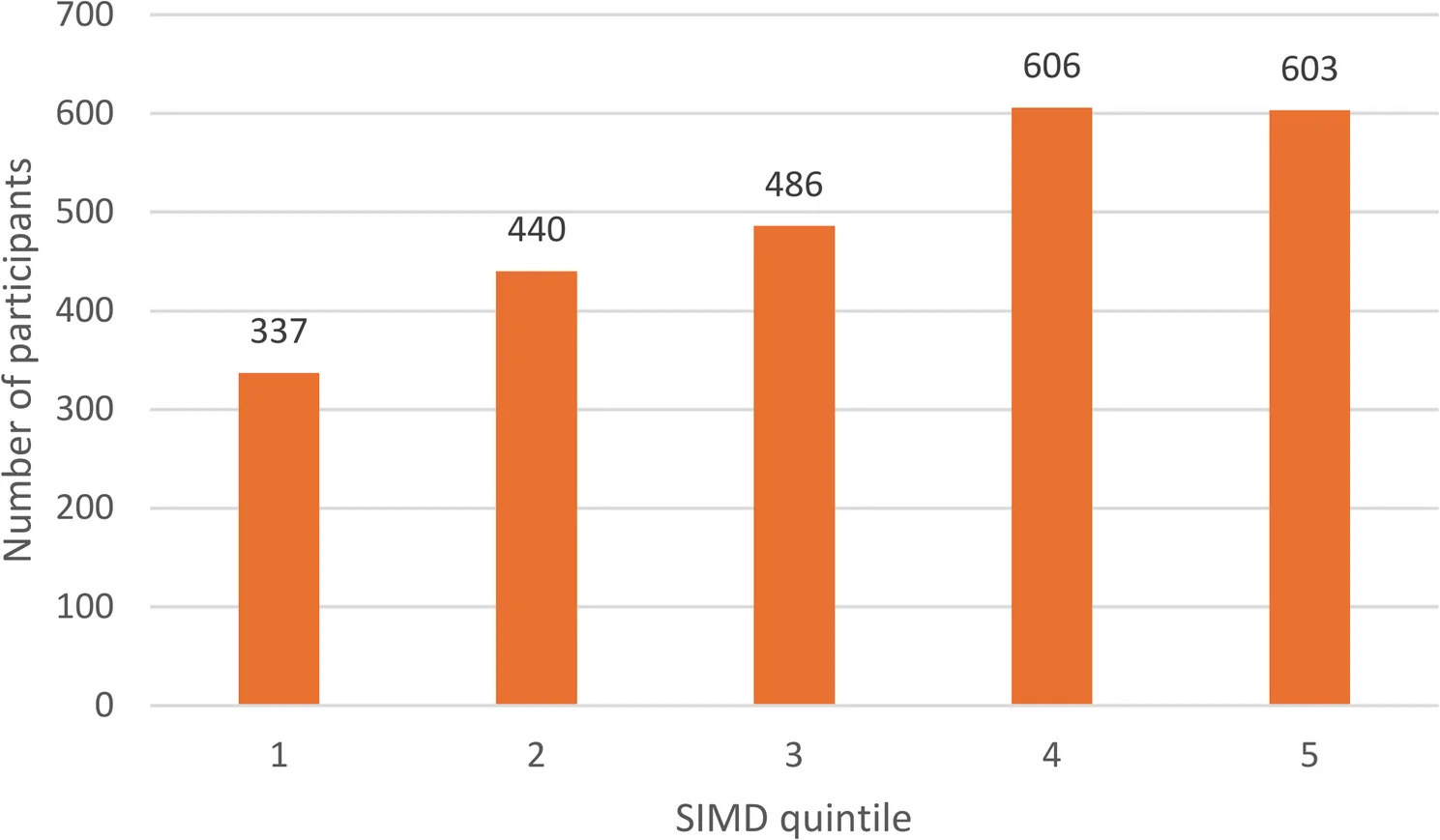
Number of participants within each SIMD quintile

The antenatal survey received 2,523 responses from 24,895 distributed surveys, a response rate of 10%. The distribution of participants across SIMD quintiles are displayed in Fig. 2. Fifty-one participants did not specify their postcode, therefore those participants data have been removed from deprivation analyses.

Seventy-six respondents (3%) were classed as having an unplanned pregnancy (LMUP 0–3), 476 (19%) were classed as ambivalent (LMUP 4–9) and 1969 (78%) classed as planned [10–12].

**Fig. 3:**
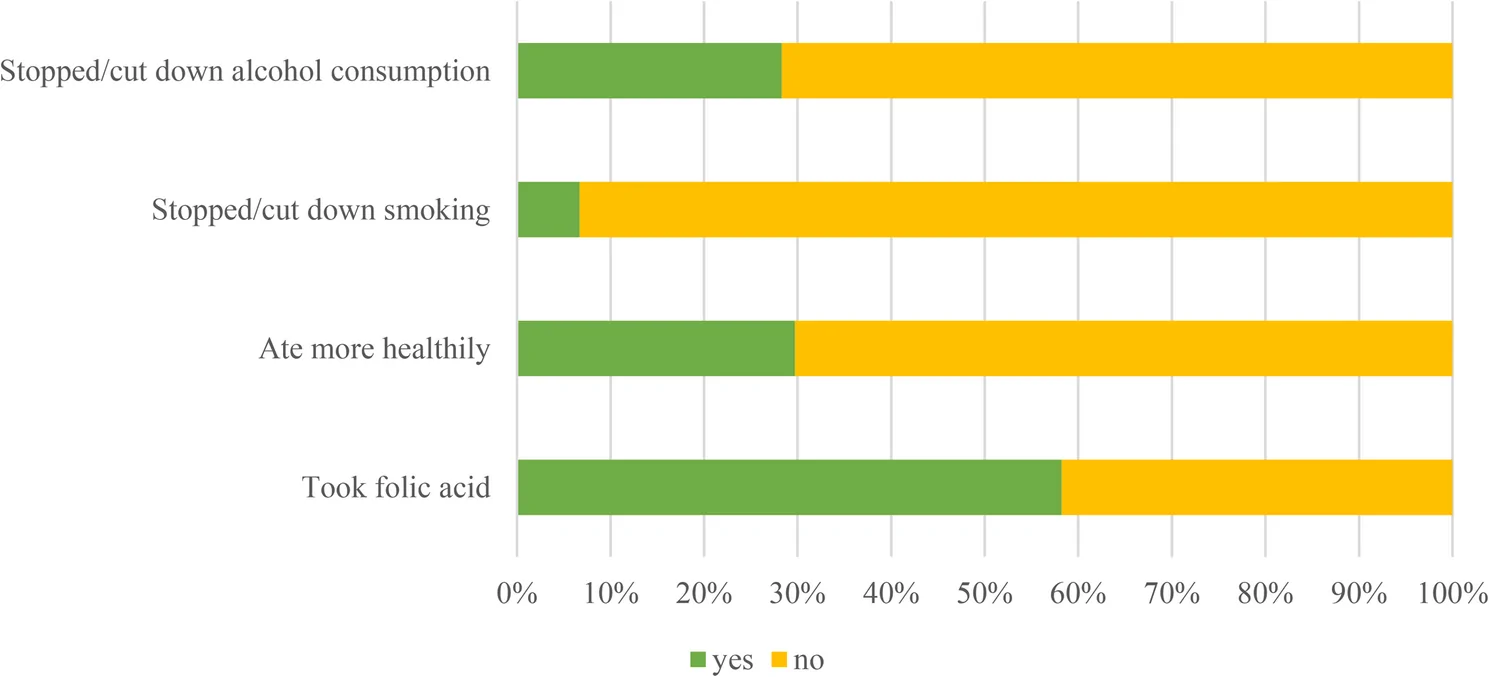
Percentage of participants who engaged in individual behaviour changes before conception

The four included behaviours (folic acid, smoking, alcohol and diet) and the percentage of participants who engaged with them is displayed in Fig. 3. The most common behaviour change was folic acid supplementation where 58.2% of participants (*N* = 1467) reported taking it before conception. This was followed by eating more healthily where 29.7% (*N* = 749) stated that they engaged in the behaviour and 70.3% (*N* = 1772) did not. 28.3% of participants (*N* = 713) stated that they reduced or stopping alcohol consumption whilst 71.7% (*N* = 1808) did not. Smoking cessation or reduction was reported by 6.7% of participants (*N* = 169) whereas 93.3% (*N* = 2352) reported that they did not. It should be noted that data regarding respondent’s history regarding any baseline smoking or alcohol consumption before pregnancy planning was not collected.

### RQ1: What is the association between deprivation and multiple behaviour change prior to conception?

**Fig. 4:**
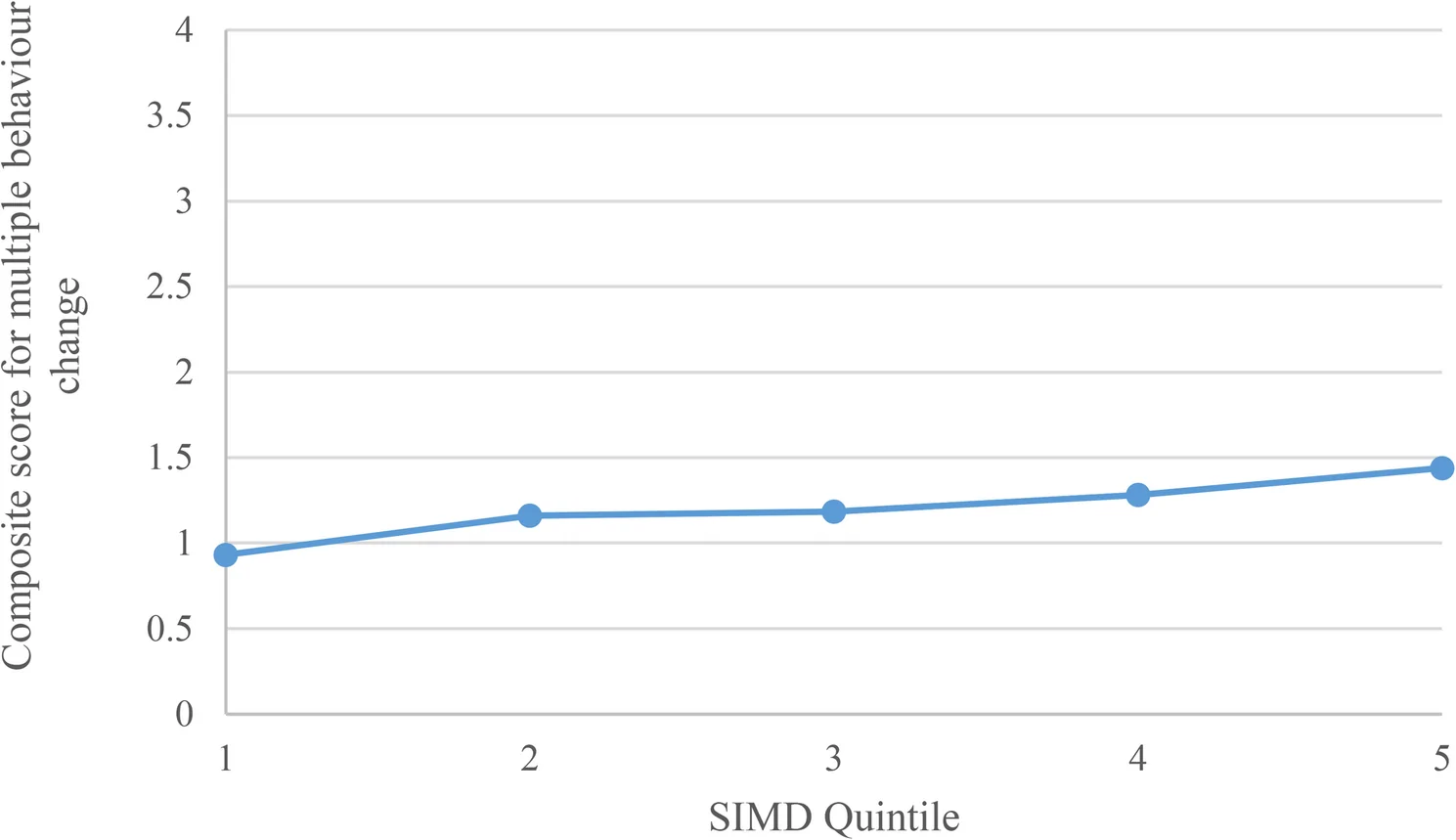
Mean score for engagement in multiple behaviour change across SIMD quintiles

Figure 4 displays the mean scores for engagement in multiple behaviour change across SIMD quintiles. A one-way ANOVA shows there was a significant effect of relative deprivation on mean composite score for behaviour change (F 4,2467 = 12.44, *p*<.05). Post hoc comparisons using the Bonferroni Adjustment indicated that the mean score for composite behaviour change in the most deprived quintile, SIMD 1, was significantly lower than every other SIMD quintile. The mean score for composite behaviour change in the least deprived quintile, SIMD 5 (was significantly higher than every other SIMD quintile with the exception of SIMD 4 The results of the one-way ANOVA are displayed in Table 1.

**Table 1:** ANOVA comparisons of multiple behaviour change across SIMD quintiles

SIMD Quintile	*n*	Mean	SD	Bonferonni Adjustment				
				SIMD 1	SIMD 2	SIMD 3	SIMD 4	SIMD 5
1	337	0.93	1.08		< 0.05	< 0.05	< 0.01	< 0.01
2	440	1.16	1.14	< 0.05				< 0.01
3	486	1.18	1.09	< 0.05				< 0.01
4	606	1.28	1.12	< 0.01				
5	603	1.44	1.44	< 0.01	< 0.01	< 0.01		

### RQ2: Is pregnancy planning associated with multiple health behaviour change prior to pregnancy?

**Fig. 5:**
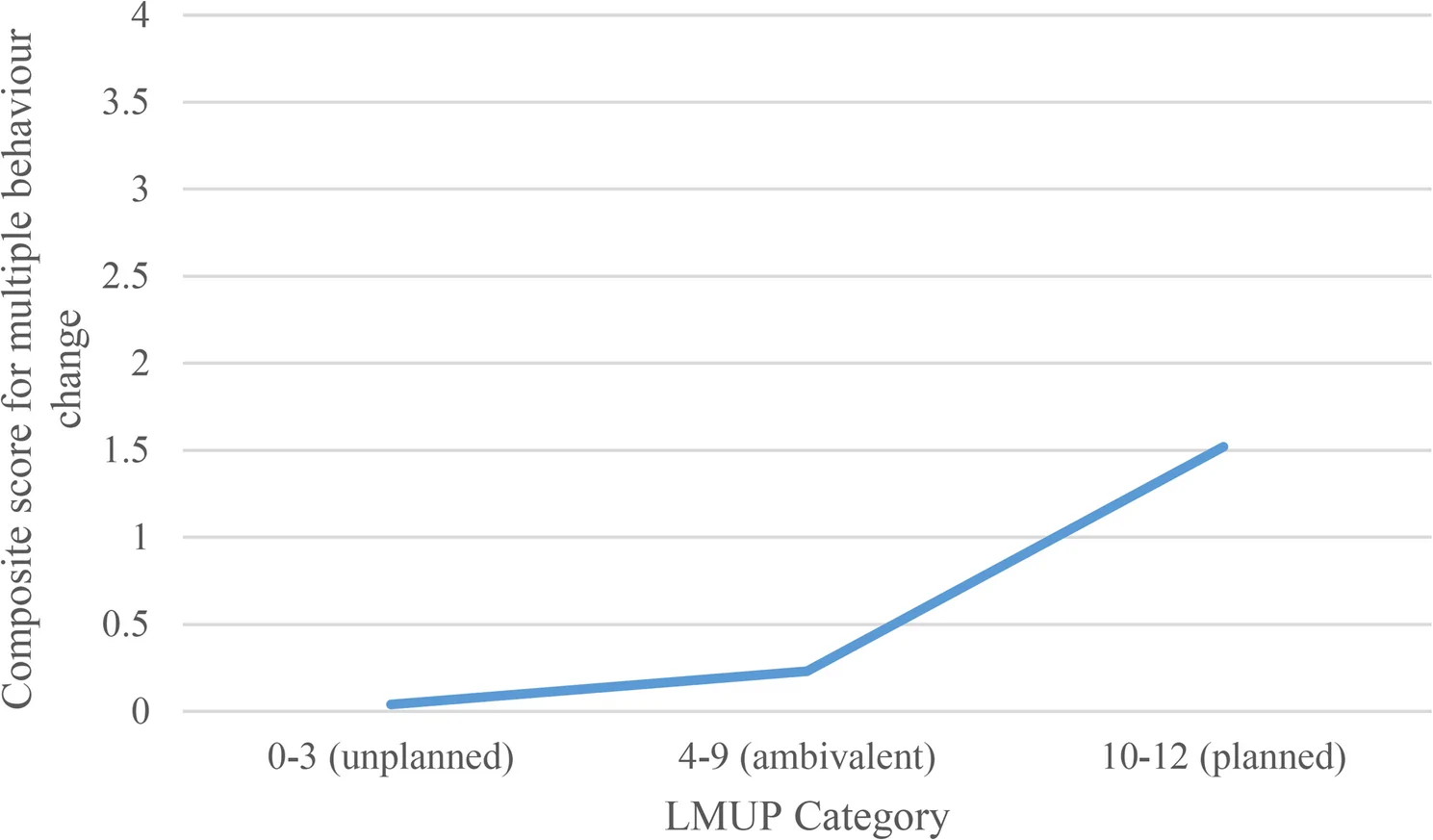
Mean score for engagement in multiple behaviour change across LMUP categories

Figure 5 displays the mean scores for engagement in multiple behaviour change across the three categories of the LMUP. A one-way ANOVA was conducted to explore the impact of LMUP category (planned, ambivalent, unplanned) on multiple behaviour change. There was a significant effect of LMUP category on mean composite score for multiple behaviour change (F 2,2518 = 393.45, *p*<.001). Post hoc comparisons using the Bonferroni adjustment identified that there was a significant effect of LMUP category on composite score for multiple behaviour change in the planned category (M = 1.5, SD = 1.06). The mean composite score for multiple behaviour change was significantly higher in the planned category than the ambivalent category (M = 0.23, SD = 0.60) and the unplanned category (M = 0.04, SD = 0.26).

### RQ3: How do psychological and behavioural pregnancy planning relate to multiple behaviour change?

There was a higher degree of psychological planning (M = 5.34, SD = 1.34) compared to behavioural planning (M = 4.78, SD = 1.34). Analysis showed that there was a significant difference between the mean scores for behavioural planning and engagement in multiple health behaviour change (F4,2517 = 1500.41, *p*=.00). Post hoc comparisons using the Bonferroni correction identified that there were significant differences between those who had a composite behaviour change score of 0 (M = 1.08, SD = 0.38) and for those who scored 1 (M = 1.69, SD= 0.19) with every other composite behaviour change score.

**Fig. 6:**
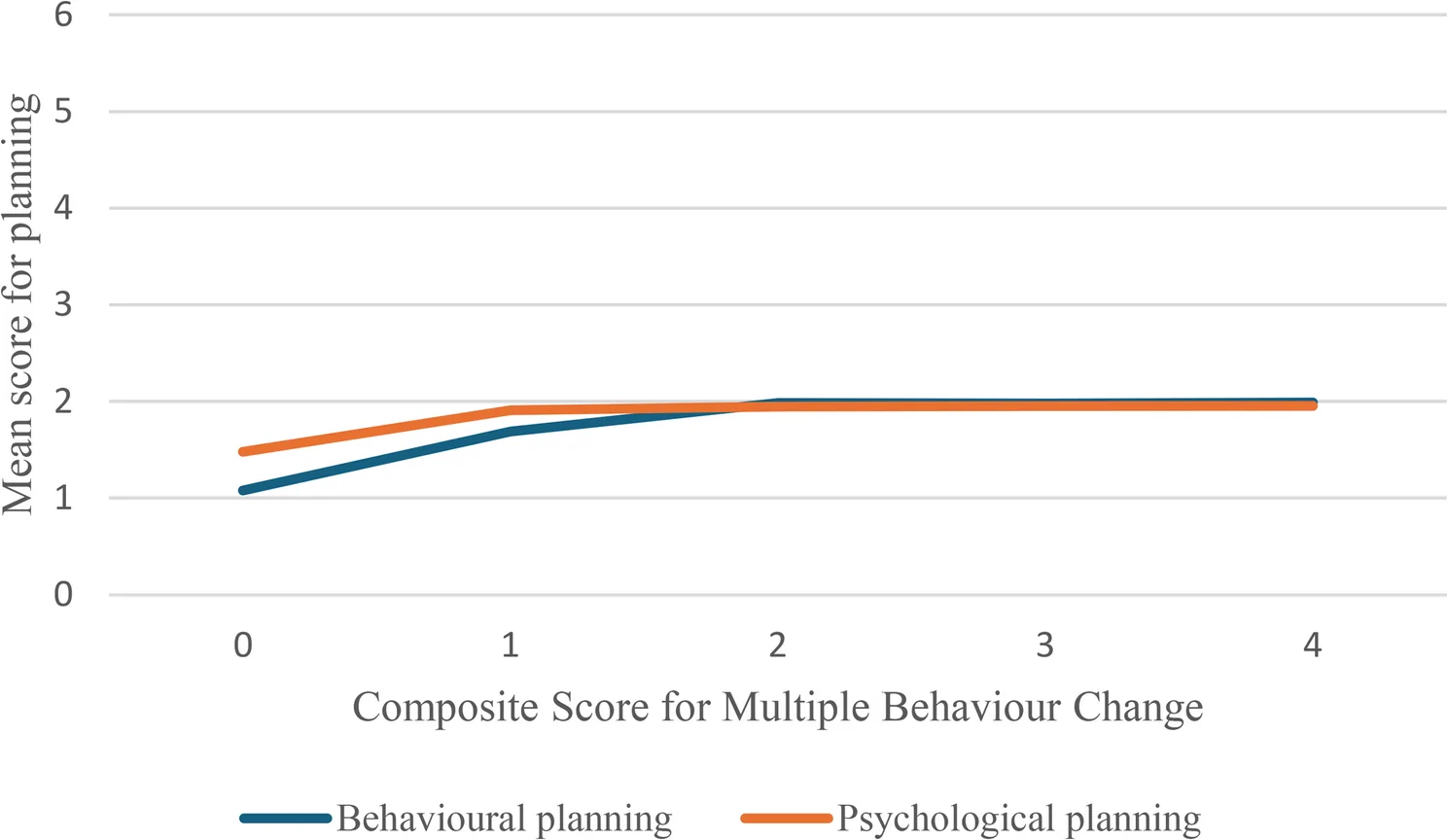
The composite score for engagement in multiple behaviour change for behavioural and psychological planning

A significant difference was also found between the mean scores for psychological planning and engagement in multiple health behaviour change (F4,2516 = 188.74, *p*=.00). Post hoc comparisons using the Bonferroni correction identified that there were significant differences between those who had a mean score of 0 for composite behaviour change (M = 1.48, SD=0.59) and every other behaviour change score. This is illustrated in Fig. 6.

## Discussion

The aim of this secondary analysis was to explore how deprivation and pregnancy planning status influence multiple behaviour change before conception.

### Deprivation and multiple behaviour change prior to conception

Living in a more deprived area of Scotland was associated with lesser engagement in multiple behaviour change prior to conception, compared to those living in the least deprived areas. This reflects the literature whereby living in economically deprived areas has been associated with reduced engagement in health behaviours such as eating healthily [19]. Children born into families living in economically deprived areas are at greater risk of comorbidities associated with poor diet quality and physical inactivity, hence perpetuating health inequalities in the next generation. The built environment and accessibility of healthy food have also been evidenced as contributing factors to low engagement in health promoting behaviours however research also considers increased stress associated with living in socio-economically deprived areas [20]. Consideration of increased exposure to stressors has raised the question as to whether risks to health among those experiencing poverty can be associated with behaviours or biological processes associated with chronic stress [21]. Whilst research acknowledges a complex interaction between factors such as a person’s environment and stress which influences engagement in health behaviours, there is limited evidence detailing why engagement in health promoting behaviours is poor in the time before conception and how preparing one’s health for pregnancy is prioritised, if at all.

### Pregnancy planning and multiple health behaviours change before conception

Those who were categorised as pregnancy planners had a significantly higher score for engagement in multiple behaviour change. Greater engagement with health behaviours such as reducing alcohol intake and smoking cessation has been reported among women planning to conceive within the next year in comparison to those whose pregnancy intentions span further than a year ahead [22]. Likewise, women who seek health advice from a medical practitioner when considering pregnancy are more likely to improve their diet and take folic acid before conception than those who had not [23]. It must be acknowledged, however, that even among the planning category, the mean composite score for multiple behaviour change was 1.5 when the maximum achievable score was 4. Whilst those planning a pregnancy engaged more in health promoting behaviour change than non-planners, this is still sub-optimal.

Our findings are also supported by Stewart and Hall [23] who showed that 49% of their female participants prepared for pregnancy, with the most common behaviour changes being eating healthier and supplementing with folic acid. From our analysis, it cannot be determined why there is a difference in engagement in multiple health behaviour change between these planning categories. However, it could be argued, as per Stewart and Hall [23], that women who plan a pregnancy are more likely to seek health information and therefore are more aware of appropriate health behaviours to prioritise such as folic acid supplementation and health eating. However along with awareness of the importance of health before conception, motivations for behaviour change may differ between those planning a pregnancy in the immediate future and those not planning. Understanding the reasons for these behavioural differences between planning categories may provide a window of opportunity for researchers to develop effective, tailored behaviour change interventions to support engagement in health promoting behaviours.

These findings align with the wider literature in which planning a pregnancy is not necessarily indicative of engagement in health promoting behaviours [24]. For example, Mass et al. [15] found that despite high scores on LMUP items one to five (indicative of pregnancy planning) as described in the current study, the majority of participants with planned pregnancies did not engage in behaviour change. Explorations into health beliefs revealed an overestimation of individual health status being associated with less of a need to prepare for pregnancy. Authors called for a public health approach to encourage engagement in health promoting behaviours before conception and reduce risks to health. This further supports the need for determining what motivates people to make changes to their health behaviours when considering a pregnancy.

### Behavioural and psychological planning with multiple behaviour change

In this sample, when the LMUP components were broken down into psychological and behavioural planning, participants engaged in a greater amount of psychological planning, compared to behavioural planning. These findings can be considered in relation to literature by Maas et al. [15] whereby the majority of their participants planned their pregnancy in a psychological sense but did not necessarily engage in health promoting behaviours before pregnancy. When both behavioural and psychological components were compared regarding their association with behaviour change, there was a significant difference between composite behaviour change scores only for those who scored zero (indicating no behavioural changes). Among those who had a composite behavioural change mean score of one or above, there were no significant differences for psychological or behavioural pregnancy planning. This reflects the literature again whereby even amongst planned pregnancies, behaviour change is not always engaged in. Goossens et al. [6] who found that despite 83% of participants planning their pregnancy, 55% engaged in alcohol consumption before conception and only 18% of the sample made efforts to improve their diet quality. These findings, similar to those of Stewart and Hall [23], accentuate the lack of awareness and prioritisation of preconception health. In an effort to support people planning to become pregnant, a life course perspective is imperative whereby preconception care could be integrated in school-based education, social media campaigns and contraceptive services to engage with women at opportune moments [24]. Additionally, preconception health offers an opportunity for policy makers to contribute to addressing health inequalities and closing the gap in health outcomes.

### Strengths, weaknesses and considerations

The large sample size of the original survey allowed a distribution of participants across the range of SIMD quintiles across Scotland. As is reflected in wider literature, participation in research is largely from people living in areas of economic advantage however this large sample size allowed comparisons to be made with those living in areas of greater deprivation. However, it would have been stronger if the response rate was higher than 10%. Due to this being a secondary analysis, the authors had no control over recruitment and response rate. Additionally, there was a rather high proportion of participants who stated their pregnancy was planned. This could reflect a self-selection bias however, it does not detract from the associations between deprivation and multiple behaviour change.

Given this is a secondary analysis, it was not possible to collect behaviour change data independent to the LMUP. Hence the data from LMUP item 6 was used both to inform the LMUP score and the behavioural change score, hence potentially inflating any association. Whilst this is a limitation and potentially confounding factor, the insights gained from this analysis offer valuable contributions to this under researched area. Furthermore, in order to adequately answer the RQs, the LMUP data was analysed in three categories (unplanned, ambivalent and planned) rather than the recommended continuous measure.

Results should be interpreted after considering the absence of baseline data measuring engagement in behaviours before women began to prepare for pregnancy. This is important for measures such as smoking and alcohol consumption as the original survey items asked women if they “stopped/cut down” on these behaviours. This has implications for the secondary analysis of the data as women did not receive a score unless they were seen to have engaged in behaviour change. This may result in women not receiving a score for reducing these behaviours not because they continued to practice them, but rather they did not smoke or drink alcohol originally and did not need to reduce or stop either behaviour. Furthermore, the original survey used retrospective self-reported data without a timeline for behaviours prior to pregnancy. Social desirability may influence responses in cases and is noted more frequently when items are measuring a sensitive topic [25]. This may be the case when addressing health behaviours such as smoking and alcohol consumption [26].

## Conclusions

This analysis identifies how women in Scotland engage in multiple behaviour change and considers the associations with deprivation and pregnancy planning status. Key findings include the low engagement in multiple behaviour change before conception, particularly among non-planners and people living in areas of higher deprivation. It is also evident that individuals psychologically prepare for pregnancy more than behaviourally prepare. When related to the wider literature it appears that planning for pregnancy and preparing for pregnancy differ and as such, it is unclear what motivates people to engage in health behaviour change before conception and research is required to explore what people understand and believe regarding the different stages of the preconception period and optimising their health before conception.

## Data Availability

The data that support the findings of this study are openly available in The UK National Archives at https://www.nationalarchives.gov.uk/doc/open-government-licence/version/3/.

## Abbreviations

ANOVA: Analysis of Variance
LMUP: London Measure of Unplanned Pregnancy
NTD: Neural Tube Defect
SIMD: Scottish Index of Multiple Deprivation
SMINS: Scottish Maternal and Infant Nutrition Survey
